# Rapamycin-insensitive mTORC1 activity controls eIF4E:4E-BP1 binding

**DOI:** 10.12688/f1000research.1-4.v1

**Published:** 2012-07-18

**Authors:** Mark Livingstone, Michael Bidinosti

**Affiliations:** 1 Biochemistry and McGill Cancer Centre, McGill University, Montreal, Canada; 2 Cytokine Signalling Unit, Institut Pasteur, Paris, France

## Abstract

The recent development of mammalian target of rapamycin (mTOR) kinase domain inhibitors and genetic dissection of rapamycin-sensitive and -insensitive mTOR protein complexes (mTORC1 and mTORC2) have revealed that phosphorylation of the mTOR substrate 4E-BP1 on amino acids Thr37 and/or Thr46 represents a rapamycin-insensitive activity of mTORC1. Despite numerous previous reports utilizing serine (Ser)-to-alanine (Ala) and threonine (Thr)-to-Ala phosphorylation site mutants of 4E-BP1 to assess which post-translational modification(s) directly regulate binding to eIF4E, an ambiguous understanding persists. This manuscript demonstrates that the initial, rapamycin-insensitive phosphorylation event at Thr46 is sufficient to prevent eIF4E:4E-BP1 binding. This finding is relevant, particularly as mTOR kinase domain inhibitors continue to be assessed for clinical efficacy, since it clarifies a difference between the action of these second-generation mTOR inhibitors and those of rapamycin analogues.

## Introduction

The mammalian target of rapamycin (mTOR) protein is an atypical Ser/Thr protein kinase named for its well-characterized inhibition by the natural product rapamycin. Rapamycin-sensitive orthologues of mTOR exist in eukaryotes from yeast to man and are required for growth and proliferation of perhaps all eukaryotic cells. As such, rapamycin has been classified as an anti-fungal agent and is clinically approved as an immunosuppressant and cancer therapy ^1– 3^. Well-characterized *in vivo* substrates of rapamycin-sensitive mTOR activity include the 70 kDa ribosomal protein S6 kinase (S6K1) and the eukaryotic [translation] initiation factor 4E (eIF4E)-binding protein 1 (4E-BP1). The mTOR-dependent phosphorylation site on S6K1, Thr389, is required for kinase activity, explaining rapamycin’s inhibition of S6K1 activity ^4^. 4E-BP1, on the other hand, is subject to multisite phosphorylation culminating in the release of bound eIF4E, leading to an ambiguous understanding of which phosphorylation site(s) regulate(s) eIF4E binding ^5^.

Mammalian 4E-BP1 is subject to an ordered phosphorylation on at least 5 major amino acid residues in response to serum-stimulation, as has been demonstrated by two-dimensional (isoelectric focusing and SDS-PAGE) electrophoresis (2DE) ^6^. This approach of separating post-translationally modified forms of a protein based on charge and apparent molecular weight has proved to be particularly useful when combined with phosphorylation-specific antibodies ^7^. Due to amino acid sequence similarity, phospho-specific anti-Thr37/46 antibodies did not allow determination of whether the initial phosphorylation event is at Thr37 or Thr46 using this technique, although priming phosphorylation at both of these sites is thought to be required for subsequent phosphorylation at Thr70, followed by phosphorylation at an unidentified site, and finally at Ser65 ^6, 8^. Given their positions flanking the amino acids responsible for eIF4E binding (amino acid residues 53–59), it is conceivable that Thr46 and Ser65 are responsible for the phosphorylation-mediated modulation of eIF4E binding occurring in response to mTOR activity. Indeed, a significant body of work supports the role of phosphorylation at Thr46 in regulating eIF4E:4E-BP1 binding ^9– 12^. Detailed analyses have led, however, to conflicting results regarding the importance of Ser65 phosphorylation in preventing this protein:protein interaction ^6, 11, 13– 15^.

While rapamycin is effective in blocking phosphorylation at Thr70 and Ser65, phospho-specific antibodies to Thr37/46 show that at least one of these sites is largely rapamycin-insensitive ^16, 17^. This residual rapamycin-insensitive phosphorylation is sensitive to serum starvation, amino acid withdrawal, and non-specific phosphatidylinositol 3-kinase (PI3K) and PI3K-like kinase (PIKK) inhibitors ^16– 19^. Furthermore, the use of mTOR kinase domain inhibitors (Torin1 and PP242) in combination with mTOR complex 2 (mTORC2)-deficient cells, has allowed the determination that Thr37/46 phosphorylation represents a rapamycin-insensitive function of mTOR complex 1 (mTORC1) ^20, 21^.

*In vivo* studies addressing the relative importance of 4E-BP1 phosphorylation sites have been hampered by its ordered phosphorylation, wherein Thr-to-Ala mutation of Thr37 or Thr46 will block subsequent phosphorylation at Thr70 and Ser65. Mounting circumstantial evidence supports the notion that phosphorylation of Thr37/Thr46 alone is the key event regulating eIF4E:4E-BP1 binding *in vivo*. Intracellular co-localization of endogenous 4E-BP1 and eIF4E best correlates with dephosphorylation at Thr37/46 ^19^. 7-methyl-GTP (cap-column) pull down of eIF4E:4E-BP1 complexes is enhanced by mTOR kinase domain inhibitors more than it is by rapamycin ^21^. Most importantly, however, mTOR active site inhibitors capable of blocking phosphorylation at Thr37/46 (and not rapamycin) induce 4E-BP-dependent phenotypes in cells ^22^.

This manuscript describes new data demonstrating that 4E-BP1 phosphorylation at the initial, mTORC1-dependent, rapamycin-insensitive phosphorylation site is alone in regulating eIF4E binding. Furthermore, this work suggests that Thr46, and not Thr37, is this key phosphorylation site. Given the recent push for pharmaceutical development of kinase inhibitors that block both the rapamycin-sensitive and rapamycin-insensitive activities of mTOR ^23^, a thorough understanding of the importance of rapamycin-insensitive mTORC1 activity is crucial. This manuscript supports the idea that clinically used mTOR kinase domain inhibitors will reduce eIF4E availability much more profoundly than have clinically approved rapamycin analogs.

## Materials and methods

Isoelectric focusing combined with SDS-PAGE based two-dimensional electrophoresis was performed as previously described ^24^. Far western analyses were performed as follows using a 32P-labelled eIF4E protein ^25^ with an N-terminal substrate peptide for heart muscle kinase (HMK). One hundred units of bovine HMK was suspended in 10 µl of 40 mM DTT and allowed to stand for 10 minutes. Five micrograms of HMK-eIF4E protein was mixed with 3 µl 10X HMK Buffer (200 mM Tris, pH 7.5, 10 mM DTT, 1 M NaCl, 120 mM MgCl2), 5ul [γ-32P] ATP 3000 Ci/mmol, 1 µl HMK (10U), and water (to 30 µl) and incubated for 45 minutes at 4°C. Probe purification was performed using Pharmacia Nick Column Sephadex G-50 DNA grade. Membranes were subjected to pre-hybridization (25 mM HEPES-KOH, pH 7.7, 25 mM NaCl, 5 mM MgCl2, 1 mM DTT, 0.1% NP40, 5% skim milk) for 5 hours at 4°C. Probe hybridization was performed in buffer (20 mM HEPES-KOH, pH 7.7; 75 mM KCl, 2.5 mM MgCl2, 0.1 mM EDTA, 1 mM DTT, 0.1% NP40, 1% skim milk) with 250,000 cpm/ml of radiolabelled probe for 10 hours at 4°C. Membranes were washed with hybridization buffer 3 times, 15 minutes prior to exposure to film (BIOMAX MS, Kodak). Following far western analysis, membranes were probed sequentially with antibodies for Phospho-Ser65, Thr70, Thr37/46, and Total 4E-BP1 (Cell Signaling Technology). Cap column based fractionation of cell lysates was performed as previously described ^26^. HeLa S3 and 293 HEK cells were treated with PP242 (2.5 µM, 30 min) or Rapamycin (10 nM, 30 min) unless otherwise indicated. Stable HeLa S3 cell lines expressing wild-type and mutant HA-4E-BP1 proteins were generated using previously described mammalian expression constructs ^8^ and G418 selection. Control siRNA and siRNA for mTOR was from Cell Signaling Technology and delivered using Lipofectamine2000 according to manufacturer’s instructions (Invitrogen). All antibodies were from Cell Signaling Technology and were used according to manufacturer’s instructions. Compounds used were rapamycin (LC Labs), PP242 (Intellikine), torin1 (Tocris), PI-103 (EMD), etoposide and nocodazole (Sigma). Standard laboratory practices were used to control bias and unwanted sources of variability in this study. The primary limitation of the datasets presented in this manuscript is that they represent single biological replicates of an experimental procedure.

## Results

### 4E-BP1 Thr37/46 Phosphorylation is sufficient to block eIF4E binding

**Figure 1.**
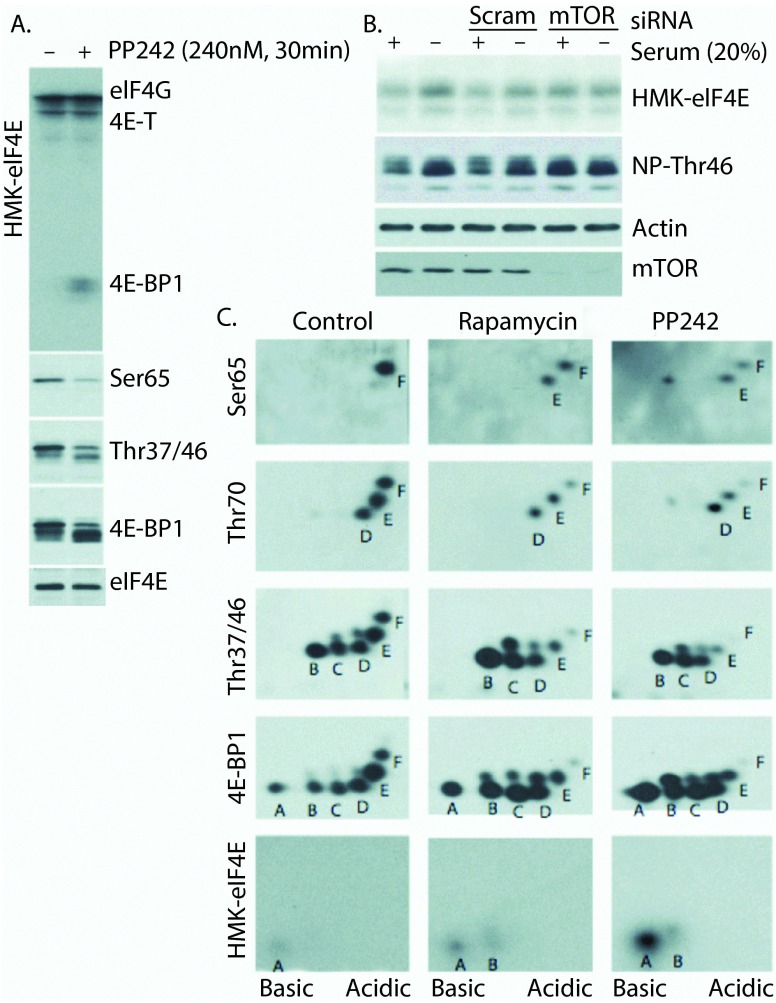
Analysis of eIF4E binding-competent forms of 4E-BP1. **A**) PP242 treatment increases the eIF4E binding ability of 4E-BP1 and reduces 4E-BP1 phosphorylation. **B**) Physiological treatment (serum starvation) modulates HMK-eIF4E binding ability of 4E-BP1 in an mTOR-dependent manner. Western blot using a non-phospho-4E-BP1/2 (Thr46) antibody parallels eIF4E binding. **C**) 2DE combined with far-western and western blot analysis to analyze HMK-eIF4E binding forms of 4E-BP1 under control (DMSO) and mTOR inhibitory (Rapamycin and PP242) conditions. Despite fairly equal abundance of spots A-F under control conditions, only "spot A" binds HMK-eIF4E. mTOR inhibition with PP242 potently increases the abundance of eIF4E-binding competent spot A, while rapamycin treatment primarily reduces phosphorylation at Ser65 (spot F).

Far western blot analysis, using radiolabeled HMK-eIF4E as a probe, is an effective measure of eIF4E-binding activity ^11, 25^. Using this approach, we demonstrate that the treatment of cells with the mTOR kinase inhibitor PP242 results in the following: (i) dramatic increases in the eIF4E-binding competent pool of 4E-BP1, (ii) reduction of 4E-BP1Thr37/46 and Ser65 phosphorylation, and (iii) unaffected binding to bands corresponding in molecular weight to 4E-T and eIF4G ( Figure 1A). The mTOR-dependent modulation of eIF4E-binding activity is also apparent under physiological conditions (serum starvation vs. serum stimulation) and can be blocked by siRNA knockdown of mTOR in HeLa S3 cells ( Figure 1B). Notably, western blot-based detection of non-phosphorylated (Thr46; denoted NP-Thr46) 4E-BP1 using a rabbit monoclonal antibody (clone: 87D12) recapitulates HMK-eIF4E binding to 4E-BP1.

To more precisely elucidate the molecular modifications of 4E-BP1 induced by PP242 that induce eIF4E binding, whole cell lysates were prepared from HEK293 cells that were subjected to short (30 min) treatment with PP242 or rapamycin followed by 2DE (isoelectric focusing and SDS-PAGE) and western blot analyses ( Figure 1C). Under control (DMSO-treated) conditions, the previously described ^6^ hierarchical, multi-site phosphorylation of 4E-BP1 is observed. Here, six differentially phosphorylated forms (labeled A-F) are detected by the total 4E-BP1 antibody, with forms B-F phosphorylated at Thr37 and/or Thr46, D-F phosphorylated at Thr70, and F phosphorylated at Ser65. Far western blot analysis demonstrates that only spot A is competent to bind HMK-eIF4E under control conditions suggesting that the modification responsible for spot B (Thr37 or Thr46) disrupts this interaction. Upon inhibition of mTOR with rapamycin or PP242, the predicted decrease in hyperphosphorylated 4E-BP1 forms is observed with an increase in hypophosphorylated 4E-BP1. While the identity of the phosphorylation event responsible for spot E remains undetermined, these data show that this phosphorylation site is resistant to mTOR inhibition, as rapamycin and PP242-resistant phospho-forms emanating from spot E appear above spots B-D. It is of note that the PP242-induced spot above spot B, which is not phosphorylated at Thr37 or Thr46, represents mono-phosphorylated 4E-BP1 (at the site responsible for spot E) and retains eIF4E-binding ability. The most likely candidates for this site are Thr84, which has shown to be responsible for a similarly slow SDS-PAGE migration ^11^, and Ser101, which has been shown to promote 4E-BP1:Raptor binding ^27, 28^. The notion that Ser101 is responsible for spot E is particularly appealing, as this would provide a sound explanation for the hierarchical ordering of this phosphorylation event prior to Ser65 (spot F). That is, it is reasonable to believe that strong 4E-BP1:Raptor binding is required for complete 4E-BP1 phosphorylation, including at Ser65.

### 4E-BP1 Thr46 is phosphorylated prior to Thr37 under normal growth conditions and prevents association with cap-bound eIF4E

**Figure 2.**
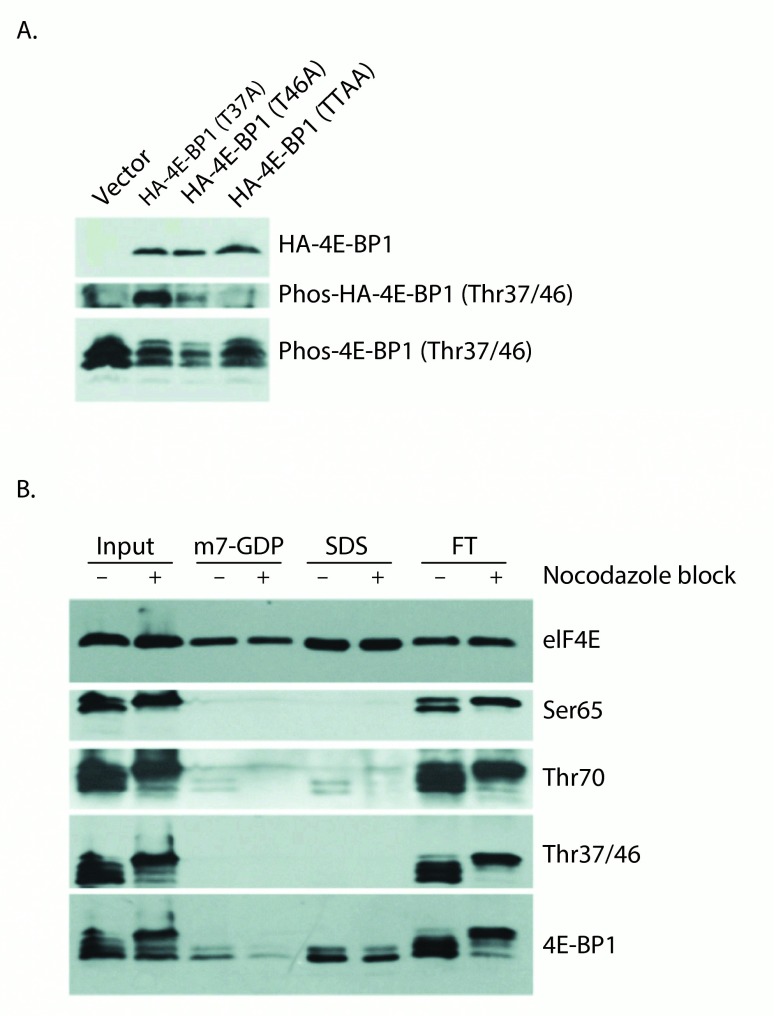
Thr46 phosphorylation is required for Thr37 phosphorylation and is sufficient to prevent eIF4E:4E-BP1 binding. **A**) HeLa S3 cells stably expressing 4E-BP1 mutant proteins were subjected to western blotting using anti-HA antibody (upper), and phospho-4E-BP1 (Thr37/46) antibody (middle and lower panels). While phospho-4E-BP1 (Thr37/46) antibodies fail to detect the Thr46Ala single and Thr37/46Ala double point mutant proteins, the Thr37Ala protein is still recognized suggesting that Thr37 is not required for Thr46 phosphorylation. Endogenous 4E-BP1 phosphorylated at Thr37/46 is shown as a control. **B**) Untreated and Nocodazole-blocked HeLa S3 cells were subjected to cap-column pull down of eIF4E and associated proteins. eIF4E was eluted with m7-GDP followed by SDS, and fractions were analyzed by western blot using eIF4E and 4E-BP1 antibodies and phospho-4E-BP1 antibodies.

Next, to determine whether Thr37 or Thr46 phosphorylation accounts for "spot B", which is impaired for eIF4E binding, HeLa cell lines stably expressing wild-type or mutant HA-4E-BP1 proteins were generated. Given the ordered phosphorylation of 4E-BP1, mutation of the primary phosphorylation site should block mutation of that of the subsequent phosphorylation site. For this reason, we analyzed Thr37Ala and Thr46Ala mutants *in vivo* using the phospho-4E-BP1 Thr37/46 antibody to determine whether the preclusion of phosphorylation at one site blocks phosphorylation at the other ( Figure 2A). While endogenous 4E-BP1 was detected by the phospho-4E-BP1 Thr37/46 antibody in lysates from all stably selected cell lines, exogenous HA-tagged 4E-BP1 was poorly detected in the Thr46Ala mutant sample, suggesting that Thr46 phosphorylation is required for subsequent Thr37 phosphorylation under normal growth conditions. These results indicate that Thr46 is the initial phosphorylation site responsible for the shift from spot A to spot B, thus phosphorylation at this site alone may be sufficient to prevent 4E-BP1 binding to eIF4E (in Figure 1C). This conclusion, that Thr46 phosphorylation precedes Thr37 phosphorylation, has previously been reached by another group ^29^.

This model, wherein 4E-BP1 phosphorylation at the initial phosphorylation site (Thr46) is sufficient to prevent eIF4E binding, is also supported by 7-methyl-GTP (cap-column) pull down data ( Figure 2B). Here the cap-column serves as a molecular mimic for the mRNA 5´-cap allowing eIF4E and associated binding proteins to be isolated from cell lysates. As a chemically induced pseudo-mitotic state has previously been shown to dramatically modulate the phosphorylation of 4E-BP1 ^26, 30^, nocodazole treatment was employed to potentially increase the diversity of 4E-BP1 phospho-forms present within our lysates. Control and nocadazole-blocked HeLa S3 cells were subjected to cap-column pull-down of eIF4E and associated 4E-BP1. This technique allowed detectable binding of only the fastest SDS-PAGE migrating forms of 4E-BP1, indicating differential binding between hypophosphorylated and hyperphosphorylated 4E-BP1 had occurred. The use of phospho-specific antibodies demonstrates that Thr37/Thr46 phosphorylated 4E-BP1 is not detectably present in this cap-column bound eIF4E fraction. Trace amounts of Thr70 phosphorylated 4E-BP1 are detected in these lanes, suggesting that mono-phosphorylated (at Thr70) 4E-BP1 exists and is eIF4E binding competent. A similar conclusion was recently reached by another group ^31^. It should be noted that the total 4E-BP1 antibody detects a doublet band in the cap-column bound eIF4E fraction. The upper band of this doublet likely represents 4E-BP1 phosphorylated at the site responsible for the above-described "spot E"; a phospho-form which retains eIF4E-binding ability.

### Existence of alternative 4E-BP1 phosphorylation patterns

**Figure 3.**
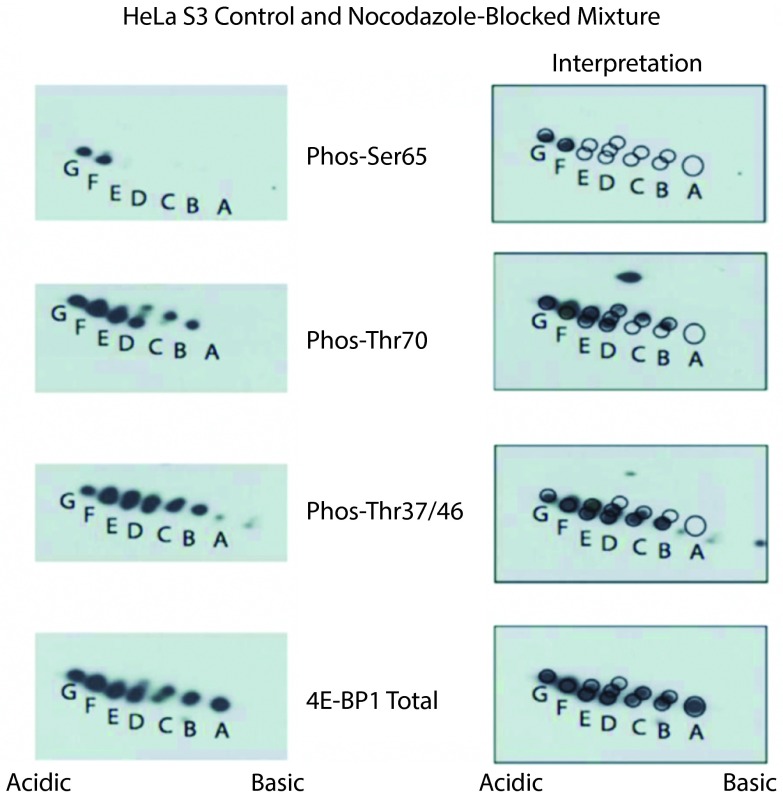
4E-BP1 phosphorylation is not strictly ordered. Untreated and nocodazole-blocked HeLa S3 cells lysed, pooled and subjected to 2D-E prior to analysis with phospho-specific and total 4E-BP1 antibodies (left panels). In addition to the standard ordered phosphorylation (Thr37 or Thr46, then "spot B", then Thr70, then "spot E", then Ser65, then "spot G"), 4E-BP1 singly phosphorylated at Thr70 is also observed indicating that this species can exist *in vivo*. To facilitate interpretation, the same images have been overlaid with a grid of circles corresponding to spots visible with the total 4E-BP1 antibody (right panels).

To further explore the potential existence of 4E-BP1 phospho-forms failing to adhere to the strict hierarchical phosphorylation pattern Thr37/46->Thr70->spot E->Ser65, untreated and nocodazole-blocked HeLa S3 cell lysates were mixed and subjected to 2D-E. Nocodazole block was again used to induce aberrant phosphorylation patterns of 4E-BP1. As shown in Figure 3, a pattern that was distinct from that produced under normal growth conditions emerged. Here, in addition to the normal hierarchical phosphorylation described above, alternative mono-phosphorylated species were present, as was an additional phosphorylation site causing the appearance of "spot G". Although, this chemical-induced atypical phosphorylation pattern may not exist under physiological conditions, these data provide support for the existence of these alternative 4E-BP1 phospho-forms.

### PP242 inhibits etoposide-induced phosphorylation of Ser/Thr-Gln motifs

**Figure 4.**
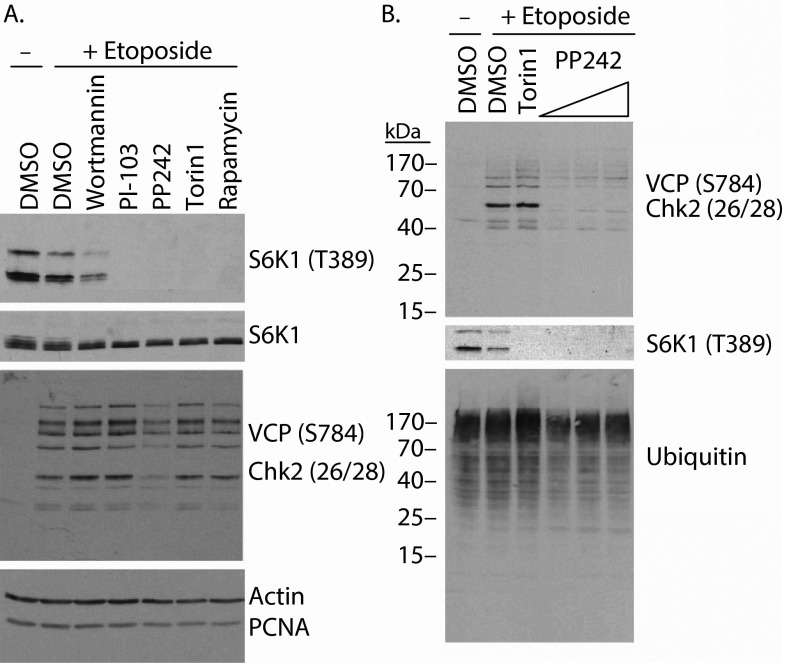
PP242 inhibits etoposide-induced phosphorylation of Ser/Thr-Gln motifs. **A**) Western blot analysis of lysates from HeLa cells pretreated with the indicated inhibitors prior to etoposide treatment reveals that PP242 reduces the appearance of multiple phospho-SQ/TQ epitopes recognized by the broadly reactive phospho-VCP/Chk2 antibody. **B**) Varying concentrations of PP242 from 0.34–1.25 µM block etoposide-induced phosphorylation of proteins recognized by the phospho-SQ/TQ antibody.

Recently it has been reported that among mTOR kinase domain inhibitors, PP242 exhibits remarkably low specificity compared with Torin1, KU63794 and WYE354 ^32^. Notably, both PP242 and Torin1 were shown to bind multiple PI3K-related kinases (PIKKs), including three key DNA damage-activated kinases ATM, ATR and DNAPK, although cell-based assays failed to show inhibition of these kinases. To assess the specificity of PP242 in our system, we employed a broadly reactive phospho-specific antibody capable of recognizing multiple phospho-Ser/Thr-Gln substrates of ATM, ATR and DNAPK ^33, 34^. Indeed, with this antibody, we observed multiple etoposide-induced bands by western blot analysis ( Figure 4), including bands corresponding to VCP phosphorylated at Ser784 (97 kDa) and Chk2 phosphorylated at Thr26/Ser28 (62 kDa). Remarkably, of the mTOR/PI3K inhibitors tested, only PP242 visibly reduced the etoposide-induced phosphorylation of these PIKK substrates ( Figure 4A). As the concentration of PP242 (2.5 µM) was higher than that used for the other inhibitors (wortmannin 0.1 µM, PI-103 1.0 µM, Torin1 0.25 µM and Rapamycin 0.01 µM), PP242 pretreatment was also assessed at 0.34, 0.68 and 1.25 µM, and gave similar results ( Figure 4B).

## Discussion

Although, our conclusion that phosphorylation at Thr46 is the key event regulating 4E-BP1:eIF4E binding has been suggested previously, none of these previous studies unambiguously established that Thr46 alone is the key important site. These studies either: (i) employed *in vivo* phosphorylation of a Thr46Ala point mutant also blocking subsequent phosphorylation events ^9, 11^; (ii) failed to identify a single important phosphorylation site ^10, 11^; or (iii) employed *in vitro* phosphorylation of Thr46Ala point mutant using a non-physiological kinase ^11, 12^.

Importantly, equally credible work has reported that phosphorylation on Thr46 is unimportant in the regulation of 4E-BP1:eIF4E binding ^8, 35^. It is interesting to note that both of these studies evaluate the capacity of *in vitro* phosphorylation at Thr46 to disrupt pre-existing 4E-BP1:eIF4E complexes and utilize cap column purification to isolate eIF4E-bound 4E-BP1. This experimental detail is particularly relevant, as it has been shown that the RNA cap can exert an allosteric effect stabilizing 4E-BP1:eIF4E binding ^14, 36^. Taken together, our data and these previous reports could suggest that Thr46 phosphorylation is sufficient to block the initial binding between eIF4E and 4E-BP1 as observed by far western analyses, but that hyperphosphorylation of 4E-BP1, including at Ser65, is required to disrupt existing 4E-BP1:eIF4E complexes.

The far western analysis of 2DE separated 4E-BP1 phospho-forms presented in Figure 1C is apparently at the limit of its useful range of detection. While this technique allows a comparison of binding efficiencies of spots A and B to evaluate the impact of Thr46 phosphorylation, it is not useful to compare the relative binding abilities of spots E and F to allow a similar assessment of the importance of Ser65 phosphorylation. Perhaps with a more sensitive assay we would have observed a similar decrease in eIF4E binding upon Ser65 phosphorylation. This would be evidence that Thr46 phosphorylation is sufficient to block the initial binding of 4E-BP1 to eIF4E when both proteins are present at low physiological concentrations, but that phosphorylation at multiple sites culminating at Ser65 is required to prevent/disrupt binding when the two proteins are present at high concentrations/local concentrations.

The data presented in Figure 2B at first seem to be at odds with this theory that 4E-BP1 phosphorylated at Thr37 or Thr46 could be pre-associated with mRNA 5´ cap-bound eIF4E in cells. This technique assesses, however, the *de novo* association of eIF4E (4E-BP1 associated or not) with an mRNA 5´ cap analog, therefore any pre-existing complexes must dissociate from cellular mRNA caps prior to isolation. These data do tell us that if there is some pool of 4E-BP1 phosphorylated on Thr46 associated with eIF4E, then this complex is not able to efficiently bind the mRNA cap analog. This assay may also evaluate the sequential binding of eIF4E to the cap followed by 4E-BP1 binding to cap-associated eIF4E and indicate in agreement with the far western data in Figure 1C that *de novo* binding is blocked by Thr46 phosphorylation.

Figure 2B and Figure 3 demonstrate that blocking cells in a pseudo-mitotic state with nocodazole results in phosphorylation of 4E-BP1 at up to 6 distinct phosphorylation sites, a finding at odds with previous work demonstrating hypophosphorylation of 4E-BP1 in mitosis ^26^. The polyclonal antibody, referred to as 11208 and used in this previous study, however, exhibits selectivity for the non-phosphorylated forms of the protein after nocodazole treatment (ML, unpublished). A similar faulty antibody-based discrepancy regarding the phosphorylation state of 4E-BP1 in meiotic oocytes was resolved ^37^. While we observed at most 6 different phospho-forms of 4E-BP1, the PhosphoSitePlus database ^38^ indicates that 20 distinct phosphorylation sites have been described in the literature and/or mass spectrometry-based datasets. This discrepancy suggests that many of these 20 phosphorylation events are either mutually exclusive, or occur only rarely, not at all, in response to specific stimuli, or in specific cell types.

Finally, the demonstration presented within this manuscript that PP242 exhibits poor specificity for mTOR in a cell-based assay for PIKK off-target effects likely does not change the conclusions of this manuscript; however, it serves as a reminder that any new mTOR inhibitor may have unanticipated effects. While the inhibition of mTORC2 by mTOR kinase domain inhibitors is expected to have profound effects, the evidence presented in this manuscript suggests that the rapamycin-insensitive activity of mTORC1 towards 4E-BP1 will also be quite important as the clinical safety and efficacy of mTOR kinase domain inhibitors is assessed.
