# Supplementary material for: Rapamycin-insensitive mTORC1 activity controls eIF4E:4E-BP1 binding
Source: F1000Res. 2012 Jul 18;1:4. [Version 1] doi: 10.12688/f1000research.1-4.v1 (PMC3782355; doi:10.12688/f1000research.1-4.v1)
Supplement: Peer review history

# Peer review: Referee response for version 1

**Published:** 2012-08-02

**Authors:** Olaf Isken, Lynne Maquat

**Affiliations:** 1 Institute of Virology and Cell Biology, University of Lübeck, Lübeck, Schleswig-Holstein, Germany; 2 Department of Biochemistry and Biophysics, University of Rochester Medical Center, Rochester, NY, USA

This submission is quite nice for experts, but not particularly well-written for those outside of the field of 4E-BP1 phosphorylation, who will have difficulty reading it ; hence my suggested clarifications below. The article is important as it demonstrates that the use of mTOR-kinase domain inhibitors would be more efficacious than the use of rapamycin analogs as therapeutics.

The work clarifies that phosphorylation of 4E-BP1 at Ser65 by mTOR is not required to inhibit 4E-BP1 binding to eIF4E and that rapamycin-insensitive mTOR-mediated phosphorylation at 4E-BP1 Thr46 is sufficient to inhibit 4E-BP1 binding to eIF4E. Phosphorylation at 4E-BP1 Thr37 is also shown to be rapamycin-insensitive. Notably, evidence that phosphorylation at Thr 37 or Thr 46 appears to be required for phosphorylation at Thr 70 and Ser 65 supported the notion that the earlier phosphorylation events were key to promoting translation.

**Improvement Suggestions:**

- Page 2, first paragraph, last sentence. The meaning of “4E-BP-dependent phenotypes” is not particularly clear.
- Page 2, line four of Materials and Methods. Shouldn’t “32″ be superscript?
- Page 2, last paragraph. The statement “Here, six differentially phosphorylated forms (A-F) …” is confusing. Is form A really phosphorylated? Earlier in the manuscript, the authors mentioned 5 major phosphorylation sites.
- In Figure 1, the assay used in this Figure requires much better description in the text. Part A of the legend requires specification of the experimental approach. Also, the title of Figure 1 could be more useful if it were informative/conclusive rather than descriptive.
- Page 3, right column, second paragraph. The sentence “While endogenous… , suggesting that Thr46 phosphorylation is required for subsequent The37 phosphorylation under normal growth conditions.” is misleading since 4E-BP1 mutant (Thr46Ala) is clearly phosphorylated at Thr37 in Fig. 2A middle panel. With the data presented here the reader is left wondering whether the differences in detecting HA-4E-BP1 (T37A) or HA-4E-BP1 (T46A) might be simply due to different affinities of the (The37/46) antibody used.
- What is the difference between the middle and the lower panel? Are the antibodies used to probe the middle and the lower panel (e.g. phosho-HA-4E-BP1 (Thr37/46) vs. phosho-4E-BP1 (Thr37/46)) different or the same? When using cell lysates of stable cell lines and a phospho-4E-BP1 (Thr37/46) antibody, shouldn’t be HA 4E-BP1 wildtype and mutant forms be detectable in the lower panel as well?
- Page 3, last paragraph, line 8. “modulate” would be clearer if described as the specific modulations that occur – in the literature a state of 4E-BP1 hypophosphorylation. The author understands that the changes are complicated and clarified from these authors’ perspective in Figure 3 and in the Discussion. However, possibly, the reader could be better informed at this point of the manuscript.
- Page 4, second paragraph, right-hand column. Please clarify the effects and usefulness of etoposide and the meaning of VCP and, in the legend, VCP/Chk2.
- In Figure 2B, the authors should provide a control western blot of an unrelated protein which does not belong to the eIF4E-associated proteins under normal conditions to show the unspecific background of this assay.It is not clear to this reader why mono-phosphorylated 4E-BP1 (at position Thr70) is detectable in the non-Nocadazole treated cell samples in Fig. 2B (lane 3 and 5) when in Fig.3 they show that mono-phosphorylation of 4E-BP1 at position Thr70 is triggered by a Nocodazol-induced block of Hela cells but not detectable under normal conditions.
- In Figure 3, why did the authors mix Control and Nocodazole-blocked Hela cell lysates instead of showing them side-by-side? Also, why are there additional spots in the interpretation panels for Phos-Thr70 and Phos-Thr37/46 that have no counterpart in the respective western blot panels? This figure is already difficult, so the authors should be careful with the overlays.
- In Figure 4, all results in this Figure are not adequately described. For example, the authors need to explain the analysis of ubiquitin and its significance to their studies.
- The sentence “ …rapamycin-insensitive phosphorylation site is alone in regulating eIF4E binding.” should read something like “ …rapamycin-insensitive phosphorylation site is alone responsible in regulating eIF4E binding”.

We have read this submission. We believe that we have an appropriate level of expertise to confirm that it is of an acceptable scientific standard.

# Peer review: Referee response for version 1

**Published:** 2012-07-30

**Authors:** Christopher Proud

**Affiliations:** 1 School of Biological Sciences, University of Southampton, Southampton, UK

I have read this submission. I believe that I have an appropriate level of expertise to confirm that it is of an acceptable scientific standard.

# Peer review: Referee response for version 1

**Published:** 2012-07-23

**Authors:** David Fruman

**Affiliations:** 1 Department of Molecular Biology and Biochemistry, University of California, Irvine, CA, USA

Through binding to eIF4E, the 4EBP proteins (4EBP1, 2, 3) interfere with formation of the eIF4F cap-binding complex. A key function of the mTOR serine-threonine kinase is to phosphorylate 4EBPs on multiple sites, which blocks their ability to bind to eIF4E thus potentiating cap-dependent translation.

Recently it has become clear that ATP-competitive mTOR kinase inhibitors are more effective than the allosteric compound rapamycin at blocking mTOR-dependent 4EBP phosphorylation, particularly on sites T37 and T46. This greater effect of mTOR kinase inhibitors on 4EBP phosphorylation correlates with increased anti-proliferative and pro-apoptotic effects. The purpose of the current study is to determine the order of phosphorylation of sites T37 and T46, and which site is most important for regulating binding of 4EBP1 to eIF4E. This is a fairly narrow question to address but the authors argue that it is important for understanding drug mechanism and that previous approaches have not addressed this question adequately.

Overall the data support the conclusion that T46 phosphorylation is the initial, rapamycin-insensitive event that blocks 4EBP1-eIF4E binding. The main approach used is two-dimensional electrophoresis (IEF followed by SDS-PAGE), then far-western blotting with labeled eIF4E to determine which spots are capable of eIF4E binding. Figure 1A provides a 1-D blot that shows convincingly that the far-western technique can reveal increased binding of eIF4E to 4EBP1 in cells treated with the mTOR kinase inhibitor PP242. Figure 1B shows that 4EBP1 phosphorylation and release from eIF4E are induced by serum in a mTOR-dependent manner. Figure 1C presents the 2D data illustrating hierarchical phosphorylation and distinct effects of rapamycin and PP242. The data support the conclusion that only a small subset of 4EBP forms are capable of binding eIF4E, that these forms lack T37/46 phosphorylation, and that PP242 increases this pool to a greater extent than rapamycin. To distinguish the roles of T37 and T46 they express HA-tagged 4EBP1 and alanine substitution mutants in HeLa cells. The data in Figure 2A show that T46A mutation, but not T37A mutation, blocks detection by the phospho-T37/46 antibody. This suggests that T46 phosphorylation precedes and is required for T37 phosphorylation. A cap binding assay in Figure 2B supports the conclusion that T37/46-phosphorylated 4EBP1 cannot bind eIF4E in the cap-binding complex, but this is not novel and the technique does not add further evidence for initial T46 phosphorylation.

The rest of the data seem added on and superfluous to the main message of the paper. The experiment in Figure 3 shows 2D far western blotting data on lysates of nocodazole-treated cells, which artificially increases the diversity of 4EBP1 spots. As the authors point out, these might not exist under physiological conditions so the relevance is unclear. Figure 4 addresses an entirely different problem, namely the lack of selectivity of the PP242 compound. While it is of some interest that PP242 differs from other compounds of this class in its apparent inhibition of DNA-PK and related kinases, this observation is too peripheral to the main study and should be developed further before publication.

I have read this submission. I believe that I have an appropriate level of expertise to confirm that it is of an acceptable scientific standard.
